# Real-world use of procalcitonin and other biomarkers among sepsis hospitalizations in the United States: A retrospective, observational study

**DOI:** 10.1371/journal.pone.0205924

**Published:** 2018-10-17

**Authors:** Eric Gluck, H. Bryant Nguyen, Kishore Yalamanchili, Margaret McCusker, Jaya Madala, Frank A. Corvino, Xuelian Zhu, Robert Balk

**Affiliations:** 1 Swedish Covenant Medical Group, Chicago, Illinois, United States of America; 2 Division of Pulmonary, Critical Care, Hyperbaric, and Sleep Medicine, Loma Linda University, Loma Linda, California, United States of America; 3 Texas Tech University Health Sciences Center, Amarillo, Texas, United States of America; 4 Diagnostics Information Solutions, Roche Diagnostics, Pleasanton, California, United States of America; 5 Genesis Research LLC, Hoboken, New Jersey, United States of America; 6 Rush University Medical Center, Chicago, Illinois, United States of America; Azienda Ospedaliero Universitaria Careggi, ITALY

## Abstract

**Background:**

Sepsis management guidelines endorse use of biomarkers to support clinical assessment and treatment decisions in septic patients. The impact of biomarkers on improving patient outcomes remains uncertain.

**Methods:**

Retrospective observational study of adult sepsis discharges between January 1, 2012, and December 31, 2015, from Premier Healthcare Database hospitals. Sepsis was defined by an All Patients Refined Diagnosis-Related Group code of 720 (septicemia and disseminated infections). Use of four biomarker strategies was evaluated based on hospital records: (i) >1 procalcitonin (PCT), (ii) 1 PCT, (iii) no PCT but ≥1 C-reactive protein (CRP) and/or lactate and (iv) no sepsis biomarkers. Associations between biomarker use and clinical and cost outcomes were examined. The primary outcome was impact of biomarker strategy on hospital costs per day.

**Results:**

Among 933,591 adult sepsis discharges during the study period, 731,392 (78%) had biomarker tests ordered. In multivariable analyses, discharges with >1 PCT had higher hospital costs per day ($1,904; 95% confidence interval [CI] $1,896–$1,911) compared with discharges with no sepsis biomarkers ($1,606; 95% CI $1,658–$1,664). Discharges with >1 PCT also had greater illness severity and antimicrobial exposure compared with other biomarker-use groups. The adjusted odds of dying during hospital stay compared with being discharged were significantly lower for sepsis discharges with >1 PCT (0.64; 95% CI 0.61–0.67) and 1 PCT (0.88; 95% CI 0.85–0.91) compared with no sepsis biomarker use. The proportion of discharges with ≥1 PCT increased almost six-fold during the study; use of other biomarkers remained constant.

**Conclusions:**

Between 2012 and 2015, PCT use among sepsis discharges increased six-fold while lactate and CRP use remained unchanged. PCT use was associated with decreased odds of in-hospital mortality but increased hospital costs per day. Serial biomarker monitoring may be associated with improved patient outcomes in the most critically ill septic patients.

## Introduction

Sepsis and septic shock remain a leading cause of death among hospitalized patients and place a large financial burden on the healthcare system [1]. In addition, the diagnosis of sepsis in patients with presumed infection is an ongoing challenge for clinicians [2]. Many rely on clinical judgment to guide care for this complex condition, with or without the use of altered vital signs and/or laboratory data. Currently, biomarkers and clinical data are combined in an attempt to improve sensitivity and specificity [3]. However, experience has shown that this approach, which features prominently systemic inflammatory response syndrome (SIRS) criteria, may not adequately distinguish whether or not the patient actually has an infection in the presence of an inflammatory state [4]. Blood cultures have also proved inadequate for differentiating infected patients from critically ill individuals who simply meet SIRS criteria [4, 5]. These diagnostic challenges may lead to the excessive or inappropriate use of empiric broad-spectrum antibiotics, which in turn carries implications for emergence of bacterial resistance, increased healthcare costs, adverse events, and *Clostridium difficile* infection [6].

The Third International Consensus Definitions for Sepsis and Septic Shock acknowledge that sepsis is a clinical diagnosis with no gold-standard diagnostic test [7]. However, sepsis management guidelines recognize the value of biomarkers in providing supplemental data that can support clinical assessment and treatment decisions [8]. Biomarker tests should focus on innate immunologic processes relating to the body’s identification of infection [9, 10]. Recent data and expert opinion suggest that the use of such biomarkers has great potential to enhance the detection of sepsis when compared with clinical judgment, SIRS, and routine laboratory data [2–4, 6, 11]. While some biomarkers can show whether a state of infection exists in a patient, others define the degree of dysregulation of the response to infection. Biomarkers may also provide benefit in directing the care of the septic patient, from identification to response to treatment. Recent guidelines and Centers for Medicare & Medicaid Services (CMS) regulations have identified procalcitonin and lactate as promising biomarkers to identify potential septic patients and to help guide antibiotic treatment and/or resuscitative efforts [12]. C-reactive protein (CRP) has also been utilized by some as a biomarker to detect the inflammatory response that occurs with sepsis.

Thus, we questioned (i) whether a biomarker-dependent diagnostic and/or therapeutic strategy has become established in the routine management of septic patients, and (ii) whether we could identify a clinical outcome or economic benefit associated with their use. The primary aim of this study was to evaluate the association between various sepsis biomarker use patterns and hospital costs per day. To accomplish this, we queried a large administrative database of hospitals with various sizes, teaching status, and geography that has been shown to be representative of the overall hospital makeup of the United States [13].

## Methods

### Study design and data source

This was a retrospective observational study of adult inpatient discharges with a discharge diagnosis of sepsis from hospitals included in the Premier Healthcare Database (PHD) [14]. The PHD contains individual-level administrative inpatient and outpatient service records, including financial data from >148 million unique patients and >700 hospitals across the United States. This database provides detailed information on clinical and hospital characteristics for a geographically diverse set of US inpatient discharges. Data are available via the PHD: https://www.premierinc.com/transforming-healthcare/healthcare-performance-improvement/premier-applied-sciences/.

The study protocol was reviewed and approved by the Institutional Review Board at Rush University Medical Center Office of Research Affairs and was determined to be exempt from the need for patient consent since it involved analysis of de-identified clinical data included in a national administrative database.

### Study population

The study included adult patients admitted into one of the Premier Healthcare member hospitals between January 1, 2012, and December 31, 2015, who had a discharge diagnosis of sepsis. Since the diagnosis of sepsis and SIRS has been controversial for many years, and the International Classification of Diseases (ICD) –9 and ICD–10 codes may not always be used to identify sepsis or septic shock discharges, we chose to use an accepted surrogate to identify sepsis discharges for our study. The PHD is an administrative database and does not permit an in-depth analysis of clinical and laboratory data to confirm the sepsis diagnosis; thus it was imperative that we utilized a definition to ensure that the study population fulfilled the selection criteria for this study. To facilitate the identification of a population for our study that was likely to meet the diagnosis of sepsis, we used the 3M All Patients Refined Diagnosis-Related Group (APR-DRG) code of 720 (septicemia and disseminated infections) [15]. The APR-DRG classification system was selected to identify sepsis cases because this system uses diagnosis and procedure information derived from medical records to classify patients into clinically meaningful groups. APR-DRG also includes four severity-of-illness and four risk-of-mortality subclasses. These subclasses enable adjustment for these factors when comparing outcomes between categories in observational studies like ours in which clinical severity of illness scores, such as Sequential Organ Failure Assessment (SOFA) [16] or Acute Physiology, Age and Chronic Health Evaluation (APACHE scores) [17], cannot be applied because the data required to calculate scores are not available. Furthermore, APR-DRG has been used successfully as a mortality risk adjustment tool and as a method to identify sepsis cases in a number of similar observational studies [18–23].

The index date was defined as the first hospital day for a sepsis-defined hospitalization. Sepsis cases were followed until discharge (including death as a reason for discharge). Discharges were grouped according to sepsis biomarker use, which was identified on the basis of charge codes for the following tests that are frequently used to evaluate infection and sepsis risk: procalcitonin (PCT), CRP, and lactate. The groups were defined as follows: (i) discharges with more than one PCT measurement during the hospital stay (>1 PCT), (ii) those with one PCT measurement (1 PCT), (iii) those with no PCT measurement, but with at least one CRP and/or lactate measurement (non-PCT), and (iv) those with no-sepsis biomarker orders (no-sepsis biomarkers). For each group, a subanalysis was conducted on discharges that included an intensive care unit (ICU) stay during hospitalization.

### Measures/Outcomes

The following patient demographics and clinical characteristics were evaluated and defined on the index date: age, sex, race, health insurance status, hospital characteristics, and admission type. The following clinical characteristics and interventions that occurred during the hospital stay were examined: APR-DRG severity of illness and APR-DRG risk of mortality classifications, charges for antimicrobial drugs commonly used to treat sepsis (S1 Table), presence of blood culture orders, use of mechanical ventilation, and use of any of the following vasopressors and/or inotropes: norepinephrine, dopamine, vasopressin, epinephrine, phenylephrine, dobutamine, or milrinone.

Sepsis biomarker utilization patterns were examined to determine the number of PCT, CRP, and/or lactate tests ordered, and the hospital day of the first physician order for each sepsis biomarker. Clinical and cost outcomes were explored, including length of stay (LOS; total and ICU-related), duration of antimicrobial use, total antimicrobial exposure (calculated as the total number of systemic antimicrobials received per day summed over the entire course of administration as defined by Balk and colleagues [13], e.g., three antimicrobials given for 7 days each = 21 days of exposure), distribution by discharge status, rate of all-cause unplanned readmission to the same hospital (among patients alive at discharge) within 30 days of discharge, and costs (total and ICU related). Costs were derived from hospital financial data submitted to PHD. Costs were calculated on a per-discharge and a per-day basis, and included the cost of hospitalization, treatment administration, and drug costs. In order to compensate for the effect of inflation over time, all costs were adjusted to first-half 2016 US dollars using the medical service component of the US Consumer Price Index [24].

### Study oversight

This study was supported by Roche Diagnostics, which purchased the data from the PHD and performed the statistical analysis. The authors attest the accuracy and completeness of the data and the analyses. All of the authors contributed in each stage of the manuscript and reviewed and approved the final manuscript prior to submission. Writing and editorial assistance were provided by Gardiner-Caldwell Communications (funded by Roche Diagnostics) under the direction and guidance of the authors.

### Statistical analysis

Baseline demographics were analyzed with descriptive statistics. Categorical variables were analyzed with frequency counts and proportions for each category, and continuous variables were summarized using means and standard deviations. Differences between the four biomarker-use groups were analyzed by one-way analysis of variance to compare means for continuous variables and by Chi-square tests for categorical variables, as appropriate.

Multivariable regression analyses were used to produce adjusted results for all outcomes of interest. Due to the skewed nature of the cost data, a generalized linear model (GLM) with a gamma distribution and a log link function was used to estimate the mean and 95% confidence intervals (CIs) for total hospital costs and hospital costs per day between the four biomarker-use groups. The “no-sepsis biomarker use” category served as the reference group. The 95% CIs of the mean difference were created using percentile bootstrapping methods with 500 replicates. For hospital LOS, duration of antimicrobial use, and total antimicrobial exposure, a GLM with a negative binomial distribution, and a log link function were used to estimate the mean for all biomarker-use groups, and mean ratios for each group compared with the no biomarker-use group (reference group) with 95% CIs. Multinomial logistic regression and simple logistic regression were used to examine discharge status and readmission, respectively. Odds ratios for each outcome were calculated for each biomarker-use group compared to the no biomarker-use group (reference group) with 95% CIs [25].

Multivariable regression analyses were adjusted for baseline demographic, hospital, and clinical characteristics. Covariates including age group, sex, primary payer, year of hospital admission, risk of mortality (APR-DRG), and hospital characteristics (hospital location, teaching status, bed size groups, and hospital census region) were included in the multivariable regression models because of their clinical importance. Pairwise correlations between all other covariates were subsequently explored. A percentile bootstrapping approach with 500 replicates was performed with random samples of the study population to generate 95% CIs for the expected mean difference between the sepsis biomarker-use groups. For highly correlated covariates (i.e., those with correlation coefficients >0.5), one covariate from the pair was excluded from the multivariable regression models. Interaction terms (age group × sex, age group × primary payer, and two-way interaction terms between hospital characteristics) were also included in the multivariable regression models. During the regression analysis, a backward selection approach was applied to select statistically significant covariates for inclusion in the final model. Covariates and interaction terms that were not statistically significant were removed from the models.

All statistical analyses were performed using SAS version 9.4 (SAS Institute, Cary, NC). *S*tatistical significance was determined by p values <0.05.

## Results

### Patient population and characteristics

Of 26,283,377 discharges in the PHD between January 1, 2012, and December 31, 2015, there were 933,591 sepsis (APR-DRG 720) discharges among adults aged ≥18 years (3.6%; 933,591/26,283,377). Thirty-nine percent of the sepsis discharges (366,569) were associated with an ICU stay (Table 1). Analyses for the subgroup of discharges that included an ICU stay are presented in S2 to S5 Tables. The demographic and hospital characteristics of the overall septic study population are presented in Table 2. Across all discharges, the mean patient age ranged from 66.5 years (no-sepsis biomarker-use group) to 67.2 years (1 PCT group), and 36%–40% of all discharges included patients with age 75 years or older (Table 2). The majority of patients were Caucasian (70%–79%) and enrolled in Medicare plans (65%–68%). The largest proportion of discharges in all categories came from the South Census region (43%–62%), which is consistent with the known distribution of hospitals that contribute data to the PHD (44% of hospitals are from the South).

**Table 1:** Cohort selection from the Premier Healthcare Database.

Selection criterion	Number of patients	%
All inpatient hospital discharges in the Premier Healthcare Database between Jan 1, 2012 and Dec 31, 2015	26,283,377	100.0
Plus assigned APR-DRG code of 720 (septicemia & disseminated Infections)	945,392	3.60
Plus age ≥18 years	937,160	3.57
Plus admission and discharge both occurred between Jan 1, 2012 and Dec 31, 2015	933,591	3.55

APR-DRG, All Patients Refined Diagnosis-Related Group.

**Table 2:** Demographic and hospital characteristics for the overall study population (N = 933,591).

Characteristic	Sepsis biomarker use category				
	>1 PCT	1 PCT	0 PCT, ≥1 CRP, and/or lactate	No sepsis biomarkers	
Number of discharges	34,245 (100)	79,234 (100)	617,913 (100)	202,199 (100)	
Mean age, years (SD)	66.7 (16.2)	67.2 (16.8)	67.2 (17.0)	66.5 (18.2)	
Age at index date (years)					
18–44	3434 (10.0)	8435 (10.7)	68,829 (11.1)	27,580 (13.6)	
45–64	10,246 (29.9)	22,259 (28.1)	173,207 (28.0)	52,726 (26.1)	
65–74	8085 (23.6)	17,859 (22.5)	131,340 (21.3)	40,208 (19.9)	
75+	12,480 (36.4)	30,681 (38.7)	244,537 (39.6)	81,685 (40.4)	
Sex					
Male	17,113 (50.0)	38,378 (48.4)	294,821 (47.7)	89,003 (44.0)	
Female	17,131 (50.0)	40,855 (51.6)	323,088 (52.3)	113,196 (56.0)	
Unknown	1 (0.0)	1 (0.0)	4 (0.0)	0	
Race					
Black	4273 (12.5)	8992 (11.4)	73,231 (11.8)	24,611 (12.2)	
White	27,156 (79.3)	60,567 (76.4)	433,681 (70.2)	148,181 (73.3)	
Other	2816 (8.2)	9675 (12.2)	111,001 (18.0)	29,407 (14.5)	
Primary health insurance payer					
Commercial	5055 (14.8)	11,663 (14.7)	88,294 (14.3)	34,551 (17.1)	
Medicare	23,303 (68.1)	54,168 (68.4)	419,121 (67.8)	130,809 (64.7)	
Medicaid	3523 (10.3)	8033 (10.1)	71,715 (11.6)	19,751 (9.8)	
Other	2364 (6.9)	5370 (6.8)	38,783 (6.3)	17,088 (8.4)	
Hospital location (US Census region)					
Northeast	1015 (3.0)	4283 (5.4)	119,025 (19.3)	28,087 (13.9)	
Midwest	5774 (16.9)	13,400 (16.9)	115,865 (18.7)	39,662 (19.6)	
South	21,170 (61.8)	46,993 (59.3)	262,801 (42.5)	107,759 (53.3)	
West	6286 (18.4)	14,558 (18.4)	120,222 (19.5)	26,691 (13.2)	
Hospital teaching status					
Teaching	13,020 (38.0)	25,420 (32.1)	261,384 (42.3)	72,269 (35.7)	
Non-teaching	21,225 (62.0)	53,814 (67.9)	356,529 (57.7)	129,930 (64.3)	
Urban or rural hospital location					
Urban	31,540 (92.1)	72,716 (91.8)	529,707 (85.7)	167,284 (82.7)	
Rural	2705 (7.9)	6518 (8.2)	88,206 (14.3)	34,915 (17.3)	
Hospital size (number of beds)					
0–99	1349 (3.9)	3226 (4.1)	33,308 (5.4)	16,505 (8.2)	
100–299	8785 (25.7)	19,495 (24.6)	232,705 (37.7)	77,943 (38.5)	
300–499	12,282 (35.9)	28,680 (36.2)	182,346 (29.5)	61,161 (30.3)	
≥500	11,829 (34.5)	27,883 (35.1)	169,554 (27.4)	46,590 (23.0)	

CRP, C-reactive protein; PCT, procalcitonin; SD, standard deviation.

Data are presented as number (%) unless stated otherwise.

Differences between groups were statistically significant (p <0.001) for all variables.

Regardless of sepsis biomarker-use category, sepsis cases were predominantly admitted to the hospital via the emergency department (78%–88%) (Table 3). Discharges with >1 PCT had a greater severity of illness (as categorized by the APR-DRG severity of illness score) compared to the other sepsis biomarker-use groups (Table 3). More than half of discharges (55%) with >1 PCT order had an extreme APR-DRG severity of illness score; 42% of discharges with 1 PCT, 40% of discharges with at least one CRP and/or lactate, and 25% of discharges with no-sepsis biomarker orders had an extreme APR-DRG severity of illness score. A similar pattern was observed for the APR-DRG risk of mortality scores.

**Table 3:** Clinical characteristics for the overall study population (N = 933,591).

Characteristic	Sepsis biomarker use category			
	>1 PCT	1 PCT	0 PCT, ≥1 CRP, and/or lactate	No sepsis biomarkers
Number of discharges	34,245 (100)	79,234 (100)	617,913 (100)	202,199 (100)
Year of hospital admission				
2012	2935 (8.6)	6991 (8.8)	125,612 (20.3)	58,894 (29.1)
2013	5496 (16.0)	13,351 (16.8)	147,875 (23.9)	53,604 (26.5)
2014	10,196 (29.8)	23,913 (30.2)	165,820 (26.8)	48,900 (24.2)
2015	15,618 (45.6)	34,979 (44.1)	178,606 (28.9)	40,801 (20.2)
Admission type				
Emergency	29,955 (87.5)	68,746 (86.8)	543,635 (88.0)	156,900 (77.6)
Trauma center/urgent	3220 (9.4)	8194 (10.3)	55,908 (9.0)	28,242 (14.0)
Elective	994 (2.9)	2071 (2.6)	16,684 (2.7)	16,058 (7.9)
Unknown	76 (0.2)	223 (0.3)	1686 (0.3)	999 (0.5)
APR-DRG severity of illness score				
Minor	264 (0.8)	1304 (1.6)	13,889 (2.3)	8320 (4.1)
Moderate	3340 (9.7)	12,119 (15.3)	108,324 (17.5)	52,716 (26.1)
Major	11,766 (34.4)	32,232 (40.7)	247,271 (40.0)	90,728 (44.9)
Extreme	18,875 (55.1)	33,579 (42.4)	248,429 (40.2)	50,435 (24.9)
APR-DRG risk of mortality score				
Minor	1760 (5.1)	6530 (8.2)	63,448 (10.3)	34,375 (17.0)
Moderate	2941 (8.6)	10,110 (12.8)	89,894 (14.5)	40,990 (20.3)
Major	9753 (28.5)	26,974 (34.0)	206,401 (33.4)	75,697 (37.4)
Extreme	19,791 (57.8)	35,620 (45.0)	258,170 (41.8)	51,137 (25.3)
Any sepsis antimicrobial use during hospital stay				
Yes	34,124 (99.6)	78,564 (99.1)	609,037 (98.6)	191,359 (94.6)
No	121 (0.4)	670 (0.9)	8876 (1.4)	10,840 (5.4)
Blood cultures ordered during hospital stay				
Yes	33,258 (97.1)	74,269 (93.7)	592,053 (95.8)	160,121 (79.2)
No	987 (2.9)	4965 (6.3)	25,860 (4.2)	42,078 (20.8)
≥1 day of mechanical ventilation during hospital stay				
Yes	9642 (28.2)	14,760 (18.6)	109,751 (17.8)	13,688 (6.8)
No	24,603 (71.8)	64,474 (81.4)	508,162 (82.2)	188,511 (93.2)
≥1 vasopressor order during hospital stay				
Yes	3427 (10.0)	5848 (7.4)	39,145 (6.3)	2763 (1.4)
No	30,818 (90.0)	73,386 (92.6)	578,768 (93.7)	199,436 (98.6)
ICU admission during hospital stay				
Yes	20,756 (60.6)	37,160 (46.9)	265,539 (43.0)	43,114 (21.3)
No	13,489 (39.4)	42,074 (53.1)	352,374 (57.0)	159,085 (78.7)

APR-DRG, All Patients Refined Diagnosis-Related Group; CRP, C-reactive protein; ICU, intensive care unit; PCT, procalcitonin.

Data are presented as number (%) unless stated otherwise.

Differences between groups were statistically significant (p <0.001) for all variables.

As would be expected, based on guidelines for sepsis management, virtually all sepsis cases received antibiotics and blood culture testing. Blood cultures were ordered less often among discharges with no sepsis biomarker use; in this group, 79% had blood cultures ordered, compared with >93% who had a blood culture ordered in all three sepsis biomarker groups. Similar results were observed for other clinical interventions associated with a more critical level of illness. Use of mechanical ventilation was less frequent among discharges with no- sepsis biomarkers use, as was administration of vasopressors/inotropes. In addition, admission to ICU was less frequent among discharges with no sepsis biomarkers use; in this group, 21% of discharges included an ICU admission compared to 43% among discharges with at least one CRP and/or lactate, 47% among discharges with 1 PCT, and 61% among discharges with >1 PCT order (Table 3).

### Sepsis biomarker utilization

Sepsis biomarkers were ordered in 731,392 (78%) of all sepsis discharges included in this study. PCT use was limited; 32,245 (3.7%) of discharges had >1 PCT and 79,234 (8.5%) had 1 PCT order during the hospital stay. CRP and lactate were used more often than PCT; 617,913 (66%) of discharges had no PCT orders but at least one CRP and/or lactate order. Among these 617,913 discharges, 96% had at least one lactate order and 17% had at least one CRP order (Table 2). Over the course of the study, the number of patients who had 1 PCT or >1 PCT determination increased from 1.5% to 5.7% and 3.5% to 13%, respectively, while the number of septic patients who had no PCT sampling but were monitored with lactate or CRP remained fairly constant (65%–67.1%). The percentage of sepsis discharges with no biomarker use decreased 50% from 30.3% to 15.1% over the 4-year study (S1 Fig).

**Fig 1:**
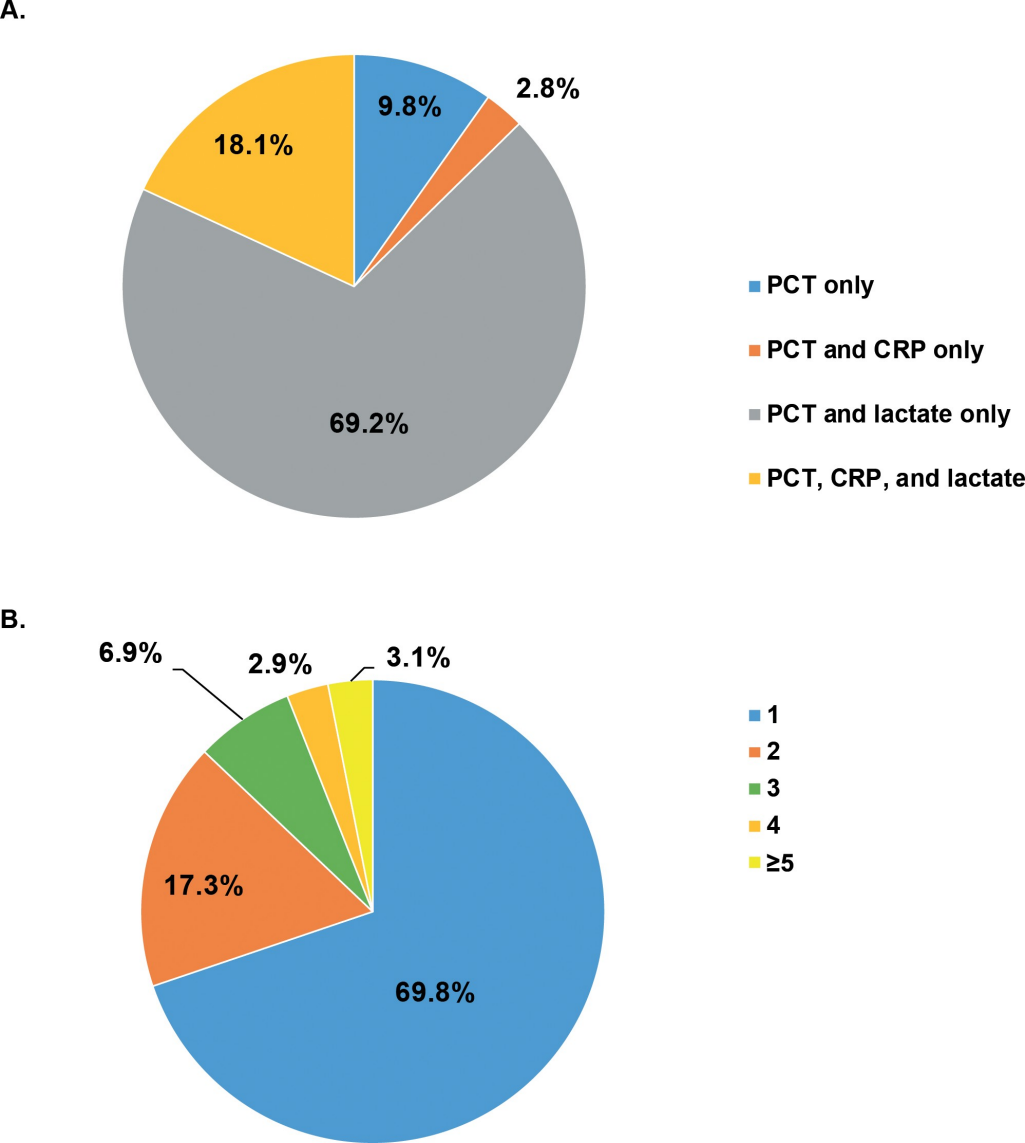
Sepsis biomarker ordering patterns among discharges with ≥1 PCT test (N = 113,479). **(A)** Patterns of sepsis biomarker orders. **(B)** Frequency of PCT test orders. CRP, C-reactive protein; PCT, procalcitonin.

**Fig 2:**
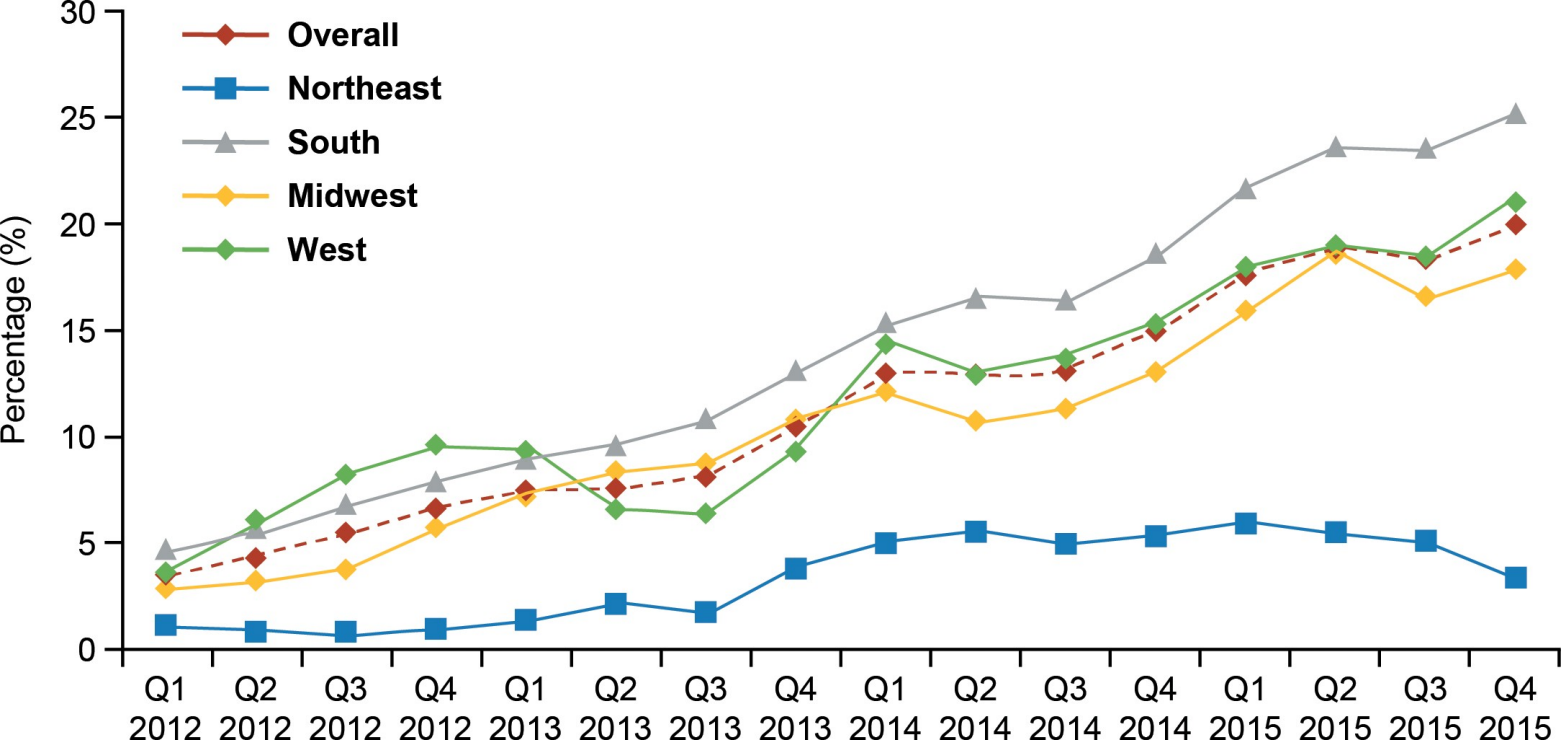
Quarterly proportion of sepsis discharges with ≥1 PCT measurement by US Census region (y-axis maximum value = 30%). PCT, procalcitonin; Q, quarter.

Among discharges with any PCT measurement, the majority also had at least one lactate measurement (Fig 1A). PCT was the only biomarker ordered in 9.8% of discharges with any PCT use. In addition, 70% of discharges with any PCT use had a single PCT order during the hospital stay and 17% had two PCT orders (Fig 1B). The proportion of discharges with ≥1 PCT measurement increased almost six-fold over the time period studied, from 3.5% in quarter (Q) 1 2012 to almost 20% in Q4 2015 (Fig 2). This increasing pattern of PCT use was observed across all regions of the United States, but at a lower rate in the Northeast Census region of the US. In the Northeast, there was a small decline in the proportion of cases with at ≥1 PCT order in Q4 2015. Additional quarters of data would be needed to determine whether this represents the beginning of a decrease in PCT use in this region or a spurious observation. Changes in the underlying hospitals that contribute data to the PHD could lead to observable differences in small categories.

### Unadjusted clinical outcomes

In unadjusted comparisons between the sepsis biomarker-use categories, mean total hospital costs, hospital costs per day, and LOS were lowest among discharges with no-sepsis biomarker use and highest among discharges with >1 PCT order (S6 Table) (p <0.01). The duration of antimicrobial use and total antimicrobial exposure were lowest among discharges with no-sepsis biomarker use and highest among discharges with >1 PCT order (p <0.01). Regardless of sepsis biomarker use, patients were most frequently discharged to home or to another healthcare facility. Inpatient mortality was highest among discharges with >1 PCT order and lowest among discharges with no sepsis biomarker use (p <0.01). The proportion of patients who were readmitted to the same hospital within 30 days after discharge was highest among discharges with >1 PCT order and lowest among discharges with no sepsis biomarker use (p <0.01). These results were similar among discharges that included an ICU admission (S3 Table).

### Multivariable analyses results

The GLM showed that sepsis biomarker use was associated with higher average total hospital costs ($13,546–$17,478 vs. $11,639 per admission; $1836–$1904 vs. $1661 per day) (Table 4) (p <0.01). Of note, discharges with >1 PCT measurement that included an ICU admission had lower mean daily ICU costs than those with 1 PCT or non-PCT sepsis biomarker use ($3545 vs. $3978 vs. $3929 per day, respectively) (S4 Table).

**Table 4:** Adjusted outcomes for sepsis discharges by biomarker use category^a^ (N = 922,594).

Outcome	Sepsis biomarker use category			
	>1 PCT	1 PCT	0 PCT, ≥1 CRP, and/or lactate	No sepsis biomarkers
Number of discharges	33,930	77,511	610,386	200,767
Total hospital costs, 2016 US$ (95% CI)				
Adjusted mean	$17,478 (17,350–17,607)	$13,915 (13,847–13,983)	$13,546 (13,522–13,569)	$11,639 (11,602–11,676)
Mean difference^b^	$5839 (5668–6006)	$2276 (2170–2381)	$1907 (1849–1966)	–
Hospital costs per day, 2016 US$ (95% CI)				
Adjusted mean	$1904 (1896–1911)	$1889 (1884–1894)	$1836 (1835–1838)	$1661 (1658–1664)
Mean difference^b^	$243 (231–256)	$228 (220–236)	$175 (171–180)	–
Overall length of hospital stay, days (95% CI)				
Adjusted mean	9.23 (9.17–9.29)	7.40 (7.36–7.43)	7.35 (7.34–7.36)	6.93 (6.91–6.95)
Mean ratio	1.33 (1.32–1.34)	1.07 (1.06–1.07)	1.06 (1.06–1.06)	–
Duration of sepsis antimicrobial use, days (95% CI)				
Adjusted mean	7.96 (7.91–8.01)	6.29 (6.26–6.32)	6.32 (6.31–6.33)	5.80 (5.78–5.82)
Mean ratio	1.37 (1.36–1.38)	1.08 (1.08–1.09)	1.09 (1.08–1.09)	–
Total antimicrobial exposure, days (95% CI)				
Adjusted mean	15.07 (14.96–15.19)	11.92 (11.86–11.98)	11.68 (11.66–11.70)	10.12 (10.09–10.16)
Mean ratio	1.49 (1.48–1.50)	1.18 (1.17–1.18)	1.15 (1.15–1.16)	–
Discharge status, OR (95% CI)				
Died in hospital	0.64 (0.61–0.67)	0.88 (0.85–0.91)	0.98 (0.96–1.00)*	–
Home	Ref	Ref	Ref	–
Hospice	0.88 (0.84–0.93)	0.94 (0.90–0.98)	1.07 (1.04–1.10)	–
Other HC facility or unknown	1.07 (1.04–1.10)	0.99 (0.97–1.02)	1.12 (1.11–1.14)	–
Patients alive at discharge readmitted to same hospital within 30 days, OR (95% CI)^c^	1.06 (1.02–1.10)	1.04 (1.01–1.07)*	1.03 (1.01–1.04)	–

CI, confidence interval; CRP, C-reactive protein; HC, healthcare; OR, odds ratio; PCT, procalcitonin.

^a^Patients with missing values or 0 for cost variables were excluded from the outcomes analysis.

^b^Versus no sepsis biomarkers (reference): 95% CIs for mean differences were calculated using bootstrapping approach (repeated 500 times).

^c^The most recent readmission within 30 days after sepsis discharge was considered. Patients with readmission on the same day as prior discharge were considered as planned readmissions and were excluded from the readmission analysis. Thus, N = 790,679.

*p >0.05; not statistically significant.

Differences between groups were statistically significant (p <0.001) for all variables, except where noted.

When compared with discharges without sepsis biomarker use, discharges with >1 PCT measurement had significantly longer LOS (both total and ICU; Table 4 and S5 Table). This concurs with the relatively high proportion of discharges (55%) with >1 PCT order who had an extreme APR-DRG severity of illness score (see above). In addition, the adjusted mean duration of antimicrobial use and total antimicrobial exposure were significantly higher among discharges with sepsis biomarker use compared to discharges with no sepsis biomarker use (Table 4). The mean ratios for both antimicrobial-use variables were highest for discharges with >1 PCT order compared with discharges with no sepsis biomarker use. Longer durations of antimicrobial use were observed in discharges in the sepsis biomarker groups relative to no sepsis biomarker use (p <0.0001).

In contrast to the unadjusted clinical outcomes, the adjusted odds of dying during a hospital stay (with being discharged to home as a reference) were significantly lower for discharges with >1 PCT order (0.64; 95% CI 0.61–0.67) and discharges with 1 PCT order (0.88; 95% CI 0.85–0.91) compared with discharges with no sepsis biomarker use, after accounting for possible confounding variables, including baseline demographic, hospital, and clinical characteristics. There was no significant reduction in the odds of dying during hospital stay in discharges with CRP and/or lactate use only when compared with discharges with no sepsis biomarker use.

The odds of readmission to the same hospital within 30 days after discharge were slightly higher for all sepsis biomarker-use groups relative to discharges with no sepsis biomarker use: >1 PCT = odds ratio (OR) 1.06 (95% CI 1.02–1.10); 1 PCT = OR 1.04 (95% CI 1.01–1.07); non-PCT = OR 1.03 (95% CI 1.01–1.04) (Table 4). Among discharges that included an ICU admission, the odds of readmission were similar among those with and without sepsis biomarker use (S4 Table).

## Discussion

Our study used a large administrative database that included APR-DRG–identified sepsis discharges from US hospitals to evaluate the impact of measuring sepsis biomarkers on hospital costs and clinical outcomes from 2012 to 2015. The study period coincided with an endorsement for PCT testing by the Surviving Sepsis Guideline and implementation of the CMS-mandated measurement of lactate levels in patients in whom sepsis was considered in the differential diagnosis or who manifested hypotension or shock [12, 26]. The CMS guideline also mandated a follow-up lactate evaluation in all patients with an initial elevation, which typically implies that those patients have a greater severity of illness compared with those without shock and/or no lactate elevation. Interestingly, the number of lactate measurements did not increase substantially over this period of study. This may reflect the typical time lag between a recommendation for care and the incorporation of those recommendations into routine care; or the fact that there were no adverse economic consequences associated with not measuring lactate. In addition, the clinical utility of lactate in the management of sepsis was already accepted prior to the period covered by our study. It is possible that compliance with use of lactate measurements would increase further once economic consequences are associated with failure to obtain such data [27].

There was a dramatic increase in the number of discharges that included single or multiple PCT measurements during the period included in our study. Moreover, the number of sepsis discharges with no biomarker measurements fell significantly, and increased utilization was not seen for CRP. This is most likely because CRP is usually viewed as a biomarker of inflammation rather than sepsis [28].

Overall, we found the ordering of >1 PCT test to be associated with greater severity of illness, and with more interventions such as admission to ICU, use of vasopressors and use of mechanical ventilation. There was a concomitant increase in LOS, total antimicrobial exposure, and hospital costs in stays that included PCT measurements. However, adjusted clinical outcomes analysis showed that the odds of inpatient death were significantly lower among hospital stays in which more than one PCT was measured (>1 PCT group) when compared with stays with no sepsis biomarker use.

These outcomes appear conflicting at first, but may simply relate to a greater baseline severity of illness among discharges with the most PCT use compared with the discharges in which no sepsis biomarkers were measured. The proportion of hospital stays that included mechanical ventilation, vasopressors, and admission to ICU was lowest among stays with no-sepsis biomarker orders, and highest among stays with >1 PCT measurement. Healthcare providers may be more likely to order biomarkers for sicker patients as a means to guide treatment decisions for these complex cases. In turn, this scenario may result in reduced mortality risk but higher total direct hospital costs compared with cases in which biomarkers are not measured. Alternatively, these observations may reflect less intensive care in less seriously ill patients, who would in turn be expected to require shorter LOS with reduced antimicrobial usage and hospital costs, but would not explain the higher mortality rate.

The association between PCT measurement and reduction of mortality in patients with sepsis has been reported by other authors. The MOSES study, which was carried out in 13 emergency departments and ICUs in the United States, showed that the use of PCT was likely to aid sepsis care [29]. Inability to decrease PCT levels by >80% was found to be an independent predictor of mortality in these patients [29]. PCT kinetics over the first 72 hours were reported to predict ICU or in-hospital mortality in another study completed in two US critical care units [30]. Conversely, a recent Cochrane Review encompassing 10 trials with 1215 patients reported no firm association between PCT guidance of antimicrobial therapy and the minimization of mortality, mechanical ventilation, clinical severity, reinfection, or duration of antimicrobial therapy [31].

Severity of illness is a common thread in the utilization of biomarkers in patients with sepsis. In this analysis, biomarker use was significantly less frequent among sepsis discharges with lower severity of illness as measured by APR-DRG scales, use of mechanical ventilation, vasopressor support, and ICU admission. Hospital LOS and antimicrobial use duration were also shorter among these discharges. On the other hand, sepsis discharges with >1 PCT order were more likely to require mechanical ventilation and vasopressor support, have increased exposure to antimicrobial therapy, and have higher readmission rates. Biomarker use among these discharges was associated with significant reductions in mortality. Also, the ICU cost per day was lower among sepsis discharges with biomarker use, which implies increased effectiveness of treatment. Sepsis severity has been highlighted elsewhere as a predictor of death in all patients receiving appropriate initial antibiotic therapy [32], and it is reasonable to hypothesize that improved biomarker assessment in these critically ill patients might be associated with optimization of therapy and improvements in survival.

Other authors have focused on the use of PCT levels in blood or PCT clearance as prognostic markers in patients with sepsis [30, 33–36]. Huang and colleagues suggested that increased PCT clearance, but not serum levels of PCT, was associated with improved survival in a small cohort (N = 48) of ICU patients with severe sepsis or septic shock [33]. In addition, a meta-analysis of 21 studies in 6007 patients with pneumonia showed elevated PCT to be linked to death from community-acquired pneumonia [34], and data from Korea have shown changes in PCT and CRP levels to be associated with outcomes in critically ill patients with sepsis [35]. Adamik and associates reported frequent endotoxemia in patients with septic shock admitted to ICU that was associated with elevated PCT and high mortality [36]. Other studies have focused on the use of PCT testing to guide antibacterial therapy [37–41]. One such study in 4507 critically ill patients showed an association between the use of PCT monitoring and reduction of duration of antibiotic treatment, together with a significant reduction in mortality [37]. In addition, a health technology assessment of 12 databases suggested cost-effectiveness and utility of PCT testing when used to guide the discontinuation of antibiotic therapy [40]. Of interest in the present context is a study in 94 patients with sepsis or septic shock in two hospitals in Brazil in which either CRP or PCT was reported to be effective for guiding therapy and reducing antibiotic use [42].

The focus of the above trials and analyses differs somewhat from the novel approach described in our study. We aimed to investigate the evolution of biomarker testing over a 4-year period in a very large retrospective US population, and to determine whether changes in biomarker testing practices were likely to influence clinical and cost outcomes in patients with sepsis. The previous studies suggest that it is reasonable to expect changing patterns of biomarker testing to be associated with effects on clinical and other outcomes.

Chu et al also used the PHD to examine PCT use among ICU admissions for sepsis during a 12-month period in 2012. The authors observed that a low proportion of cases had serial PCT measurements ordered during the hospital stay. Similar to the results of our study, Chu et al did not observe an association between use of PCT measurements and decreased antibiotic use. In contrast to our study, they did not detect any decrease in mortality associated with PCT use. One possible explanation for this difference in findings could be that we included an adjustment for severity of illness in our study, while Chu et al did not have an equivalent covariate included in their analysis. The authors hypothesized that PCT may be more effective when used conjunction with an algorithm to guide implementation [43].

The design of this study comparing four biomarker-use strategies in a large administrative database made it difficult to fully control for underlying differences in patient characteristics between the groups. Analyses that include only two comparators, such as those that compare cases with any biomarker use versus none, and that use statistical methods such as propensity score matching to control for differences in patient and clinical characteristics may be better able to examine differences in these outcomes. An analysis in >33,000 PCT-managed and > 98,000 non–PCT-managed ICU patients that used propensity scores showed significantly lower hospital and ICU LOS, and reduced total, ICU, and pharmacy costs when PCT testing on the first day of ICU admission was used [13].

In the current study, we chose to use traditional multivariable analysis methods instead of propensity score matching for three reasons. First, due to the large sample size in our study, we were able to generate reliable and statistically efficient estimates for multiple outcomes with robust regression models based on different sets of statistically significant confounding variables. Second, traditional multivariable analysis methods were able to provide a predictive model between the outcome variable and the confounding variables, which offers detailed insights into which covariates have the strongest influence on the outcome [44]. Finally, propensity score matching between four comparator groups would have resulted in large information loss from the analysis, and consequent loss of precision for parameter estimation and generalizability of the results. Propensity score matching is a well-established analysis method for comparisons between two groups, but has not been routinely used in analyses with three or more comparator groups [45]. Therefore, we chose to use traditional multivariable analysis methods.

An expected limitation of this study is its retrospective design. Patients who were deemed to be irretrievably ill might have been excluded from having any biomarker measurements, thus making mortality risk appear more favorable for the biomarker group. The large and diverse population studied here minimizes this likelihood, but such an effect is nevertheless still possible. Our analysis also included sepsis discharges that did not include an ICU stay. This approach may have resulted in the inclusion of patients with less severe disease and may partly explain the lower costs and shorter LOS among discharges without biomarker use, but would not be expected to have an associated higher mortality rate. The use of APR-DRG code 720 (septicemia and disseminated infections) to select probable sepsis cases may have resulted in the inclusion of discharges without sepsis, but it is difficult to use any discharge code to ensure our study population reflects sepsis as defined in the new Sepsis-3 definition [7]. Since we had an administrative database that did not include in-depth clinical details, using an accepted sepsis detection tool, APR-DRG, also allowed us to adjust for severity of illness in the multivariable analyses. It is possible that misclassification of non-sepsis cases as sepsis in this analysis may have limited our ability to compare outcomes across the biomarker-use groups because non-sepsis cases would be unlikely to have these biomarkers measured.

Another limitation of our study is the lack of information to explain why sepsis biomarkers tests were ordered. The PHD includes billing information for the clinical care that is provided during the course of a hospital stay, but does not included detailed clinical notes that could provide insights into why specific tests were ordered or how a patient’s clinical course evolved over time. Therefore, we were able to observe an association between the use of these biomarkers and our outcomes of interest, but cannot infer a causal association between biomarker use and clinical outcomes.

Two additional limitations for this study should be noted. First, the results of our analysis should not be generalized to countries other than the United States. Differences in clinical practice patterns and health care financing and delivery in other countries are likely to impact the associations observed in our study. Second, the administrative nature of the data we used in this analysis preclude the ability to derived clinical conclusions from the data. Analyses of medical record data or prospective studies that collect detailed clinical information would be better suited to address clinical questions about the impact of sepsis biomarkers.

## Conclusion

We have demonstrated in this large, retrospective analysis that from 2012–2015, there was an increase use of biomarkers among sepsis hospital discharges in the United States. The use of lactate and CRP remained constant over this time, but there was a six-fold increase in the use of PCT. In addition, PCT use was associated with a decreased odds of in-hospital mortality and increased antimicrobial use, LOS, and hospital costs. These results suggest that there has been an increase in the use of biomarkers to help management of septic patients, and this guidance may be associated with improvements in patient outcome in the most critically ill sepsis cases.

## Supporting information

S1 Table Antimicrobial drugs commonly used to treat sepsis. (DOCX)

S2 Table Demographic characteristics for discharges that included an ICU stay (N = 366,569). (DOCX)

S3 Table Clinical characteristics for discharges that included an ICU stay (N = 366,569). (DOCX)

S4 Table Unadjusted clinical outcomes for sepsis discharges by biomarker-use category for discharges that included an ICU staya (N = 361,863). (DOCX)

S5 Table Adjusted outcomes for sepsis discharges by biomarker-use category for discharges that included an ICU staya (N = 361,863). (DOCX)

S6 Table Unadjusted outcomes for sepsis discharges by biomarker-use categorya (N = 922,594). (DOCX)

S1 Fig Sepsis biomarker use over time among sepsis discharges. (**A**) Change over time proportion of discharges between all sepsis biomarker-use categories. (**B**) Zoomed view from (**B**) showing 1 and >1 PCT and 0 PCT, CRP, or lactate categories; note that y-axis maximum = 35%. CRP, C-reactive protein; PCT, procalcitonin.
(PDF)
