# Supplementary material for: Real-world use of procalcitonin and other biomarkers among sepsis hospitalizations in the United States: A retrospective, observational study
Source: PLoS One. 2018 Oct 17;13(10):e0205924. doi: 10.1371/journal.pone.0205924 (PMC6192638; doi:10.1371/journal.pone.0205924)
Supplement: S1 Table — (DOCX) [file pone.0205924.s001.docx]

**S1 Table.** **Antimicrobial drugs commonly used to treat sepsis.**

| **Antifungals** | |
| --- | --- |
| Amphotericin b | Ketoconazole |
| Anidulafungin | Micafungin |
| Caspofungin | Pentamidine |
| Fluconazole |  |
| **Antibacterials** | |
| Amikacin | Gentamicin |
| Ampicillin/sulbactam | Imipenem |
| Ampicillin | Kanamycin |
| Azithromycin | Levofloxacin |
| Aztreonam | Lincomycin |
| Cefamandole | Linezolid |
| Cefazolin | Meropenem |
| Cefepime | Metronidazole |
| Cefotaxime | Mezlocillin |
| Cefotetan | Minocycline |
| Cefoxitin | Moxifloxacin |
| Ceftaroline | Nafcillin |
| Ceftazidime | Neomycin |
| Ceftizoxime | Ofloxacin |
| Ceftolozane/tazobactam | Oxacillin |
| Ceftriaxone | Penicillin |
| Cefuroxime | Piperacillin |
| Cephradine | Piperacillin/tazobactam |
| Chloramphenicol | Quinupristin/dalfopristin (Synercid) |
| Ciprofloxacin | Rifampin |
| Clindamycin | Tedixolid/tedizolid |
| Colistimethate (Colistin) | Telavancin |
| Dalbavancin | Ticarcillin |
| Daptomycin | Ticarcillin/clavulanate potassium |
| Doripenem | Tigecycline |
| Doxycycline | Tobramycin |
| Ertapenem | Trimethoprim-sulfamethoxazole |
| Erythromycin | Vancomycin |
