# Supplementary material for: Real-world use of procalcitonin and other biomarkers among sepsis hospitalizations in the United States: A retrospective, observational study
Source: PLoS One. 2018 Oct 17;13(10):e0205924. doi: 10.1371/journal.pone.0205924 (PMC6192638; doi:10.1371/journal.pone.0205924)
Supplement: S2 Table — (DOCX) [file pone.0205924.s002.docx]

**S2 Table.** **Demographic characteristics for discharges that included an ICU stay (N = 366,569).**

| **Characteristic** | **Sepsis biomarker use category** | | | | | |
| --- | --- | --- | --- | --- | --- | --- |
|  | **>1 PCT** | **1 PCT** | **0 PCT, ≥1 CRP, and/or lactate** | **No sepsis biomarkers** | | |
| Number of discharges | 20,756 (100) | 37,160 (100) | 265,539 (100) | 43,114 (100) | | |
| Mean age, years (SD) | 66.4 (15.5) | 67.3 (15.6) | 67.3 (15.7) | 67.7 (16.0) | | |
| Age at index date (years) | | | | | | |
| 18–44 | 1879 (9.1) | 3192 (8.6) | 23,133 (8.7) | 3893 (9.0) | | |
| 45–64 | 6680 (32.2) | 11,334 (30.5) | 81,540 (30.7) | 12,389 (28.8) | | |
| 65–74 | 5219 (25.1) | 9050 (24.3) | 62,399 (23.5) | 9918 (23.0) | | |
| 75+ | 6978 (33.6) | 13,584 (36.6) | 98,467 (37.1) | 16,914 (39.2) | | |
| Sex |  |  |  |  | | |
| Male | 10,476 (50.5) | 18,523 (49.8) | 130,030 (49.0) | 20,359 (47.2) | | |
| Female | 10,279 (49.5) | 18,636 (50.2) | 135,506 (51.0) | 22,755 (52.8) | | |
| Unknown | <5 | <5 | <5 | 0 | | |
| Race |  |  |  |  | |  |
| Black | 2775 (13.4) | 4527 (12.2) | 32,255 (12.2) | 5141 (11.9) | |  |
| White | 16,291 (78.5) | 28,001 (75.3) | 189,355 (71.3) | 32,158 (74.6) | |  |
| Other | 1690 (8.1) | 4632 (12.5) | 43,929 (16.5) | 5815 (13.5) | |  |
| Primary health insurance payer | | | | | | |
| Commercial | 2897 (14.0) | 5029 (13.5) | 34,975 (13.2) | 5919 (13.7) | | |
| Medicare | 14,189 (68.4) | 25,818 (69.5) | 183,550 (69.1) | 30,387 (70.5) | | |
| Medicaid | 2199 (10.6) | 3831 (10.3) | 31,116 (11.7) | 4151 (9.6) | | |
| Other | 1471 (7.1) | 2482 (6.7) | 15,898 (6.0) | 2657 (6.2) | | |
| Hospital location (US Census region) | | | | | | |
| Northeast | 607 (2.9) | 1731 (4.7) | 40,767 (15.3) | | 3837 (8.9) | |
| Midwest | 3282 (15.8) | 5868 (15.8) | 53,004 (20.0) | | 8831 (20.5) | |
| South | 13,928 (67.1) | 23,125 (62.2) | 123,457 (46.5) | | 24,931 (57.8) | |
| West | 2939 (14.2) | 6436 (17.3) | 48,311 (18.2) | | 5515 (12.8) | |
| Hospital teaching status | | | | | | |
| Teaching | 8570 (41.3) | 12,922 (34.8) | 106,300 (40.0) | | 12,645 (29.3) | |
| Non-teaching | 12,186 (58.7) | 24,238 (65.2) | 159,239 (60.0) | | 30,469 (70.7) | |
| Urban or rural hospital location | | | | | | |
| Urban | 19,366 (93.3) | 34,310 (92.3) | 226,059 (85.1) | | 34,095 (79.1) | |
| Rural | 1390 (6.7) | 2850 (7.7) | 39,480 (14.9) | | 9,019 (20.9) | |
| Hospital size (number of beds) | | | | | | |
| 0–99 | 767 (3.7) | 1330 (3.6) | 14,047 (5.3) | 4133 (9.6) | | |
| 100–299 | 4952 (23.9) | 9078 (24.4) | 99,826 (37.6) | 16,805 (39.0) | | |
| 300–499 | 7831 (37.7) | 14,036 (37.8) | 79,945 (30.1) | 12,795 (29.7) | | |
| ≥500 | 7206 (34.7) | 12,716 (34.2) | 71,721 (27.0) | 9381 (21.8) | | |

CRP, C-reactive protein; ICU, intensive care unit; PCT, procalcitonin; SD, standard deviation.

Data are presented as number (%) unless stated otherwise.

Differences between groups were statistically significant (p <0.001) for all variables.
