# Supplementary material for: Real-world use of procalcitonin and other biomarkers among sepsis hospitalizations in the United States: A retrospective, observational study
Source: PLoS One. 2018 Oct 17;13(10):e0205924. doi: 10.1371/journal.pone.0205924 (PMC6192638; doi:10.1371/journal.pone.0205924)
Supplement: S3 Table — (DOCX) [file pone.0205924.s003.docx]

**S3 Table.** **Clinical characteristics for discharges that included an ICU stay (N = 366,569).**

| **Characteristic** | **Sepsis biomarker use category** | | | | | | |
| --- | --- | --- | --- | --- | --- | --- | --- |
|  | **>1 PCT** | **1 PCT** | **0 PCT, ≥1 CRP, and/or lactate** | | | **No sepsis biomarkers** | |
| Number of discharges | 20,756 (100) | 37,160 (100) | 265,539 (100) | | | 43,114 (100) | |
| Year of hospital admission | | | | | | | |
| 2012 | 1940 (9.3) | 3925 (10.6) | 61,741 (23.3) | | | 14,045 (32.6) | |
| 2013 | 3616 (17.4) | 6985 (18.8) | 68,149 (25.7) | | | 11,640 (27.0) | |
| 2014 | 6446 (31.1) | 11,380 (30.6) | 69,183 (26.0) | | | 9993 (23.2) | |
| 2015 | 8754 (42.2) | 14,870 (40.0) | 66,466 (25.0) | | | 7436 (17.2) | |
| Admission type | | | | | | | |
| Emergency | 18,104 (87.2) | 32,139 (86.5) | 230,372 (86.8) | | | 33,315 (77.3) | |
| Trauma center/urgent | 1978 (9.5) | 3800 (10.2) | 25,980 (9.8) | | | 6471 (15.0) | |
| Elective | 621 (3.0) | 1088 (2.9) | 8311 (3.1) | | | 3112 (7.2) | |
| Unknown | 53 (0.3) | 133 (0.4) | 876 (0.3) | | | 216 (0.5) | |
| APR-DRG severity of illness score | | | | | | |  |
| Minor | 31 (0.2) | 112 (0.3) | 1043 (0.4) | | | 301 (0.7) |  |
| Moderate | 551 (2.7) | 1715 (4.6) | 13,575 (5.1) | | | 3664 (8.5) |  |
| Major | 5033 (24.2) | 11,097 (29.9) | 77,890 (29.3) | | | 15,977 (37.1) |  |
| Extreme | 15,141 (72.9) | 24,236 (65.2) | 173,031 (65.2) | | | 23,172 (53.7) |  |
| APR-DRG risk of mortality score | | | | | | | |
| Minor | 252 (1.2) | 743 (2.0) | 6568 (2.5) | | | 1832 (4.3) | |
| Moderate | 674 (3.3) | 1813 (4.9) | 14,264 (5.4) | | | 3845 (8.9) | |
| Major | 3784 (18.2) | 8382 (22.5) | 61,012 (23.0) | | | 13,507 (31.3) | |
| Extreme | 16,046 (77.3) | 26,222 (70.6) | 183,695 (69.1) | | | 23,930 (55.5) | |
| Any sepsis antimicrobial use during hospital stay | | | | | | | |
| Yes | 20,716 (99.8) | 36,918 (99.3) | | 261,598 (98.5) | | 41,503 (96.3) | |
| No | 40 (0.2) | 242 (0.7) | | 3941 (1.5) | | 1611 (3.7) | |
| Blood cultures ordered during hospital stay | | | | | | | |
| Yes | 20,245 (97.5) | 34,760 (93.5) | | 256,584 (96.6) | | 36,363 (84.3) | |
| No | 511 (2.5) | 2400 (6.5) | | 8955 (3.4) | | 6751 (15.7) | |
| ≥1 day of mechanical ventilation during hospital stay | | | | | | | |
| Yes | 9057 (43.6) | 13,463 (36.2) | | 98,006 (36.9) | | 11,081 (25.7) | |
| No | 11,699 (56.4) | 23,697 (63.8) | | 167,533 (63.1) | | 32,033 (74.3) | |
| ≥1 vasopressor order during hospital stay | | | | | | | |
| Yes | 3268 (15.7) | 5442 (14.6) | 36,881 (13.9) | | 2307 (5.3) | | |
| No | 17,488 (84.3) | 31,718 (85.4) | 228,658 (86.1) | | 40,807 (94.7) | | |

APR-DRG, All Patients Refined Diagnosis-Related Group; CRP, C-reactive protein; ICU, intensive care unit; PCT, procalcitonin; SD, standard deviation.

Data are presented as number (%) unless stated otherwise.

Differences between groups were statistically significant (p <0.001) for all variables.
