# Supplementary material for: Real-world use of procalcitonin and other biomarkers among sepsis hospitalizations in the United States: A retrospective, observational study
Source: PLoS One. 2018 Oct 17;13(10):e0205924. doi: 10.1371/journal.pone.0205924 (PMC6192638; doi:10.1371/journal.pone.0205924)
Supplement: S4 Table — (DOCX) [file pone.0205924.s004.docx]

**S4 Table.** **Unadjusted clinical outcomes for sepsis discharges by biomarker-use category for discharges that included an ICU stay^a^ (N = 361,863).**

| **Outcome** | **Sepsis biomarker use category** | | | | | | | |
| --- | --- | --- | --- | --- | --- | --- | --- | --- |
|  | **>1 PCT** | | **1 PCT** | **0 PCT, ≥1 CRP, and/or lactate** | | | **No sepsis biomarkers** | |
| Number of discharges | 20,591 | | 36,222 | 262,243 | | | 42,807 | |
| Mean total hospital costs (SD) | $29,832 (30,699) | | $22,153 (23,780) | $23,099 (23,447) | | | $17,629 (16,726) | |
| Mean hospital costs per day, 2016 US$ (SD) | $2436 (1662) | | $2421 (1578) | $2479 (1604) | | | $2078 (1030) | |
| Mean overall length of hospital stay, days (SD) | 12.2 (10.7) | | 9.4 (7.7) | 9.4 (7.5) | | | 8.4 (6.4) | |
| Mean total ICU costs, 2016 US$^b^ (SD) | $19,837 (24,610) | | $14,673 (18,330) | $15,251 (17,857) | | | $11,270 (12,563) | |
| Mean ICU costs per day, 2016 US$^b^ (SD) | $3545 (2931) | | $3978 (4551) | $3929 (3841) | | | $3271 (3138) | |
| Mean ICU length of hospital stay, days^b^ (SD) | 5.8 (6.8) | | 4.2 (4.7) | 4.3 (4.4) | | | 3.8 (3.8) | |
| Mean duration of sepsis antimicrobial use, days (SD) | 10.2 (7.4) | | 7.7 (6.0) | 7.8 (5.9) | | | 6.9 (5.2) | |
| Mean total antimicrobial exposure, days (SD) | 20.6 (16.2) | | 15.6 (13.1) | 15.5 (13.1) | | | 13.0 (11.5) |  |
| Discharge status, n (%) | | | | | | | | |
| Died in hospital | 4056 (19.7) | 8104 (22.4) | | 57,657 (22.0) | | 7642 (17.9) | | |
| Home | 7565 (36.8) | 13,747 (38.0) | | 94,813 (36.1) | | 17,820 (41.6) | | |
| Hospice | 1757 (8.5) | 2989 (8.3) | | 20,712 (7.9) | | 3382 (7.9) | | |
| Other HC facility or unknown | 7211 (35.0) | 11,372 (31.3) | | 89,029 (34.0) | | 13,958 (32.6) | | |
| 30-day readmission to same hospital (among patients alive at discharge), n (%)^c^ | | | | | | | | |
| Total | 16,068 (100) | | 27,187 (100) | | 196,707 (100) | | 33,876 (100) | |
| Yes | 2681 (16.7) | | 4502 (16.6) | | 32,711 (16.6) | | 5432 (16.0) | |
| No | 13,387 (83.3) | | 22,685 (83.4) | | 163,996 (83.4) | | 28,444 (84.0) | |

CRP, C-reactive protein; HC, healthcare; ICU, intensive care unit; PCT, procalcitonin; SD, standard deviation.

^a^Patients with missing values or 0 for hospital cost variables were excluded from the outcomes analysis.

^b^Patients with missing values or 0 for ICU cost variables were excluded from the outcomes analysis. N = 381,814 for these outcomes.

^c^The most recent readmission within 30 days after sepsis discharge was considered. Patients readmitted on the same day as prior discharge were considered as planned readmissions and were excluded from the readmission analysis. Thus, total N = 273,880.

Differences between groups were statistically significant (p <0.001) for all variables, except for 30-day readmission (p = 0.054).
