# Supplementary material for: Real-world use of procalcitonin and other biomarkers among sepsis hospitalizations in the United States: A retrospective, observational study
Source: PLoS One. 2018 Oct 17;13(10):e0205924. doi: 10.1371/journal.pone.0205924 (PMC6192638; doi:10.1371/journal.pone.0205924)
Supplement: S5 Table — (DOCX) [file pone.0205924.s005.docx]

**S5 Table.** **Adjusted outcomes for sepsis discharges by biomarker-use category for discharges that included an ICU stay^a^ (N = 361,863).**

| **Outcome** | **Sepsis biomarker use category** | | | |
| --- | --- | --- | --- | --- |
|  | **>1 PCT** | **1 PCT** | **0 PCT, ≥1 CRP, and/or lactate** | **No sepsis biomarkers** |
| Number of discharges | 20,591 | 36,222 | 262,243 | 42,807 |
| Total hospital costs, 2016 US$ (95% CI) |  |  |  |  |
| Adjusted mean | $26,558  (26,307–26,811) | $21,354  (21,201–21,508) | $20,920  (20,865–20,975) | $19,028  (18,901–19,155) |
| Mean difference^b^ | $7530 (7188–7919) | $2326 (2072–2560) | $1892 (1702–2052) | – |
| Hospital costs per day, 2016 US$ (95% CI) |  |  |  |  |
| Adjusted mean | $2361 (2350–2373) | $2404 (2395–2413) | $2364 (2361–2367) | $2194 (2186–2201) |
| Mean difference^b^ | $167 (147–193) | $210 (193–227) | $170 (158–180) | – |
| Overall length of hospital stay, days (95% CI) |  |  |  |  |
| Adjusted mean | 11.51 (11.41–11.61) | 9.17 (9.11–9.23) | 9.08 (9.06–9.11) | 8.78 (8.72–8.84) |
| Mean ratio | 1.31 (1.30–1.33) | 1.04 (1.04–1.05) | 1.04 (1.03–1.04) | – |

| Duration of sepsis antimicrobial use, days (95% CI) |  |  |  |  |
| --- | --- | --- | --- | --- |
| Adjusted mean | 9.74 (9.66–9.83) | 7.64 (7.59–7.70) | 7.61 (7.59–7.62) | 7.23 (7.18–7.27) |
| Mean ratio | 1.35 (1.33–1.36) | 1.06 (1.05–1.07) | 1.05 (1.05–1.06) | – |
| Total antimicrobial exposure, days (95% CI) |  |  |  |  |
| Adjusted mean | 19.46 (19.27–19.65) | 15.40 (15.29–15.52) | 14.97 (14.92–15.01) | 13.58 (13.48–13.68) |
| Mean ratio | 1.43 (1.42–1.45) | 1.13 (1.12–1.15) | 1.10 (1.09–1.11) | – |
| Total ICU costs, 2016 $US (95% CI)^c^ |  |  |  |  |
| Adjusted mean | $15,785  (15,624–15,948) | $13,140  (13,039–13,242) | $13,113  (13,076–13,150) | $12,052  (11,966–12,139) |
| Mean difference^2^ | $3733 (3507–3967) | $1088 (905–1283) | $1061 (934–1171) | – |
| ICU costs per day, 2016 US$ (95% CI)^c^ |  |  |  |  |
| Adjusted mean | $3564 (3541–3588) | $4041 (4021–4061) | $3755 (3748–3761) | $3440 (3424–3456) |
| Mean difference^b^ | $124 (82–184) | $601 (544–659) | $315 (282–350) | – |
| ICU length of stay, days (95% CI)^c^ |  |  |  |  |
| Adjusted mean | 4.84 (4.79–4.89) | 3.83 (3.80–3.86) | 3.99 (3.98–4.00) | 4.01 (3.98–4.04) |
| Mean ratio | 1.21 (1.19–1.22) | 0.96 (0.94–0.97) | 0.99 (0.99–1.00) | – |
| Discharge status, OR (95% CI) |  |  |  |  |
| Died in hospital | 0.64 (0.61–0.68) | 0.87 (0.83–0.92) | 0.95 (0.92–0.98) | – |
| Home | Ref | Ref | Ref | – |
| Hospice | 0.88 (0.82–0.95) | 0.91 (0.86–0.97) | 0.99 (0.96–1.04)^*^ | – |
| Other HC facility or unknown | 1.04 (0.99–1.00)^*^ | 0.97 (0.93–1.01)^*^ | 1.06 (1.04–1.09) | – |
| Patients alive at discharge readmitted to same hospital within 30 days, OR (95% CI)^d^ | 0.98 (0.93–1.03)^*^ | 0.98 (0.94–1.03)^*^ | 0.98 (0.95–1.01)^*^ | – |

CI, confidence interval; CRP, C-reactive protein; HC, healthcare; ICU, intensive care unit; OR, odds ratio; PCT, procalcitonin.

^a^Patients with missing values or 0 for hospital cost variables were excluded from the outcomes analysis.

^b^Versus no sepsis biomarkers (reference): 95% CIs for mean differences were calculated using bootstrapping approach (repeated 500 times).

^c^Patients with missing values or 0 for ICU cost variables were excluded from the outcomes analysis. N = 381,814 for these outcomes.

^d^The most recent readmission within 30 days after sepsis discharge was considered. Patients readmitted on the same day as prior discharge were considered planned readmissions and were excluded from the readmission analysis. Thus, total N = 273,880.

*p >0.05; not statistically significant.

Differences between groups were statistically significant (p <0.01) for all variables; except where noted.
