# Supplementary material for: Real-world use of procalcitonin and other biomarkers among sepsis hospitalizations in the United States: A retrospective, observational study
Source: PLoS One. 2018 Oct 17;13(10):e0205924. doi: 10.1371/journal.pone.0205924 (PMC6192638; doi:10.1371/journal.pone.0205924)
Supplement: S6 Table — (DOCX) [file pone.0205924.s006.docx]

**S6 Table.** **Unadjusted outcomes for sepsis discharges by biomarker-use category^a^ (N = 922,594).**

| **Outcome** | **Sepsis biomarker use category** | | | | |
| --- | --- | --- | --- | --- | --- |
|  | **>1 PCT** | **1 PCT** | **0 PCT, ≥1 CRP and/or lactate** | **No sepsis biomarkers** | |
| Number of discharges | 33,930 | 77,511 | 610,386 | 200,767 | |
| Mean total hospital costs, 2016 US$ (SD) | $23,438 (26,495) | $15,901 (18,939) | $16,072 (18,689) | $10,465 (11,353) | |
| Mean hospital costs per day (SD) | $2130 (1442) | $1969 (1311) | $1972 (1324) | $1531 (844) | |
| Mean overall length of hospital stay, days (SD) | 10.6 (9.2) | 7.8 (6.4) | 7.8 (6.3) | 6.5 (5.7) | |
| Mean duration of sepsis antimicrobial use, days (SD) | 8.9 (6.6) | 6.6 (5.1) | 6.6 (5.1) | 5.4 (4.1) | |
| Mean total antimicrobial exposure, days (SD) | 17.7 (14.5) | 12.9 (11.1) | 12.5 (11.0) | 9.4 (8.6) |  |
| Discharge status, n (%) | | | | | |
| Died in hospital | 4638 (13.7) | 10,381 (13.4) | 77,596 (12.7) | 18,699 (9.3) | |
| Home | 15,731 (46.4) | 39,560 (51.0) | 304,443 (49.9) | 116,989 (58.3) | |
| Hospice | 2438 (7.2) | 5236 (6.8) | 39,371 (6.4) | 11,535 (5.7) | |
| Other HC facility or unknown | 11,123 (32.8) | 22,334 (28.8) | 188,976 (31.0) | 53,544 (26.7) | |
| 30-day readmission to same hospital (among patients alive at discharge), n (%)^b^ | | | | | |
| Total | 28,635 (100) | 65,624 (100) | 518,572 (100) | 177,848 (100) | |
| Yes | 4465 (15.6) | 9694 (14.8) | 75,762 (14.6) | 23,179 (13.0) | |
| No | 24,170 (84.4) | 55,930 (85.2) | 442,810 (85.4) | 154,669 (87.0) | |

CRP, C-reactive protein; HC, healthcare; PCT, procalcitonin; SD, standard deviation.

^a^Patients with missing values or 0 for cost variables were excluded from the outcomes analysis.

^b^The most recent readmission within 30 days after sepsis discharge was considered. Patients with readmission on the same day as prior discharge were considered planned readmissions and were excluded from the readmission analysis. Thus, N = 790,679Differences between groups were statistically significant (p <0.001) for all variables, except where noted.
