# Supplementary figures and images for: Real-world use of procalcitonin and other biomarkers among sepsis hospitalizations in the United States: A retrospective, observational study
Source: PLoS One. 2018 Oct 17;13(10):e0205924. doi: 10.1371/journal.pone.0205924 (PMC6192638; doi:10.1371/journal.pone.0205924)

**S1 FIG**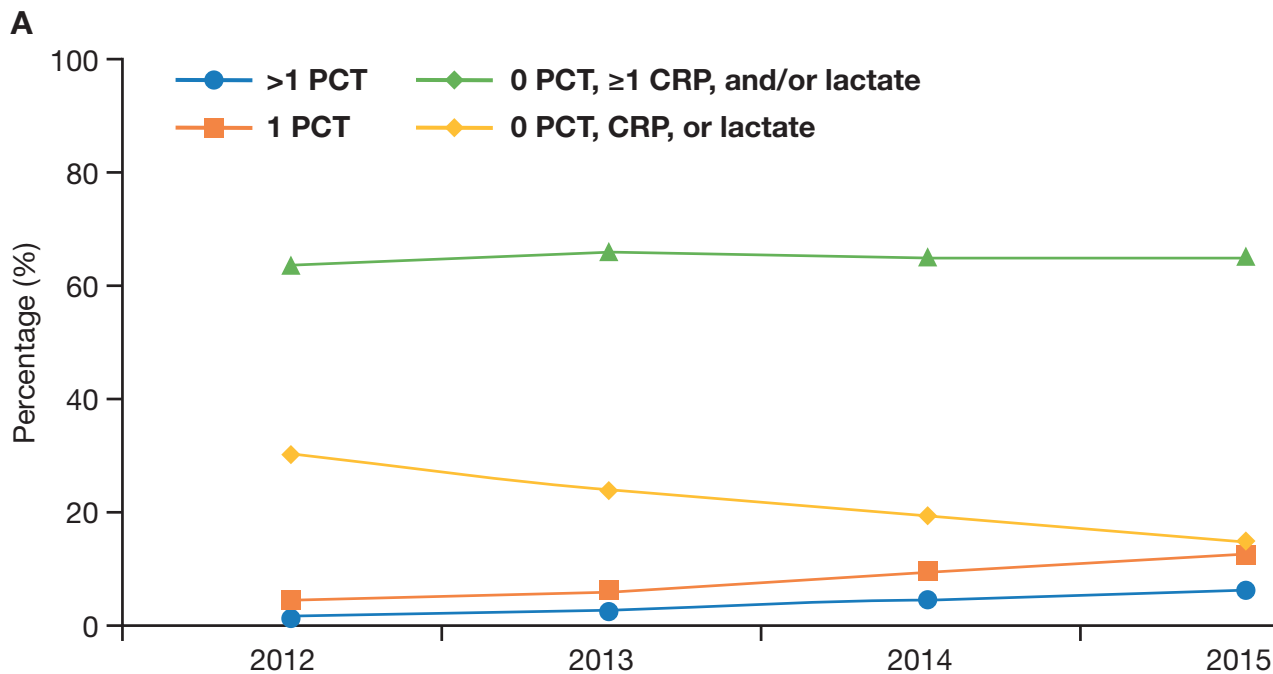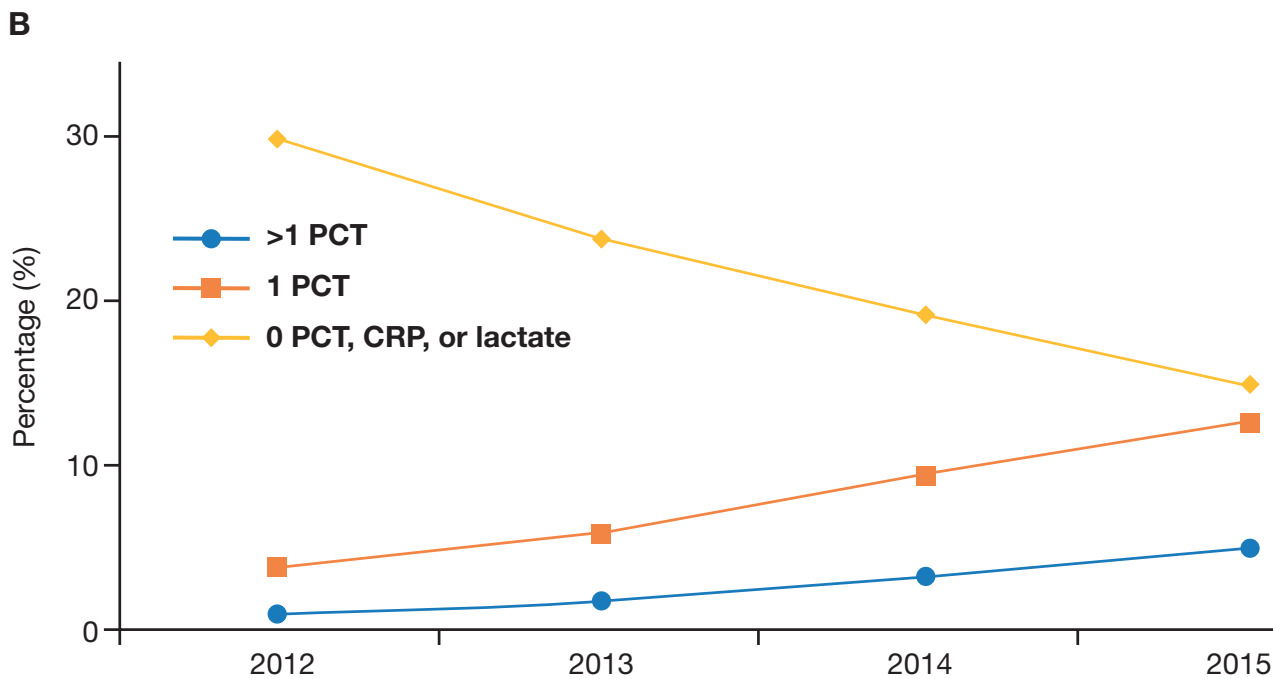

Supplement: S1 Fig — (A) Change over time proportion of discharges between all sepsis biomarker-use categories. (B) Zoomed view from (B) showing 1 and >1 PCT and 0 PCT, CRP, or lactate categories; note that y-axis maximum = 35%. CRP, C-reactive protein; PCT, procalcitonin. (PDF) [file pone.0205924.s007.pdf]
